# Transforming growth factor‐β1 signalling triggers vascular endothelial growth factor resistance and monocyte dysfunction in type 2 diabetes mellitus

**DOI:** 10.1111/jcmm.16543

**Published:** 2021-05-04

**Authors:** Lena‐Maria Makowski, Merle Leffers, Johannes Waltenberger, Evangelia Pardali

**Affiliations:** ^1^ Department of Cardiology I—Coronary and Peripheral Vascular Disease, Heart Failure University Hospital Münster, Cardiolology Münster Germany; ^2^ Cells‐in‐Motion Cluster of Excellence (EXC 1003—CiM) University of Münster Münster Germany; ^3^ Department of Cardiovascular Medicine Medical Faculty University of Münster Münster Germany

**Keywords:** hyperglycaemia, monocytes, TGF‐β, type 2 diabetes mellitus, VEGFA

## Abstract

Type 2 diabetes mellitus (T2DM) leads to monocyte dysfunction associated with atherogenesis and defective arteriogenesis. Transforming growth factor (TGF)‐β1, placenta growth factor (PlGF)‐1 and vascular endothelial growth factor (VEGF)A play important roles in atherogenesis and arteriogenesis. VEGF‐receptor (VEGFR)‐mediated monocyte migration is inhibited in T2DM (VEGFA resistance), while TGF‐β1‐induced monocyte migration is fully functional. Therefore, we hypothesize that TGF‐β antagonises the VEGFA responses in human monocytes. We demonstrate that monocytes from T2DM patients have an increased migratory response towards low concentrations of TGF‐β1, while PlGF‐1/VEGFA responses are mitigated. Mechanistically, this is due to increased expression of type II TGF‐β receptor in monocytes under high‐glucose conditions and increased expression of soluble (s)VEGFR1, which is known to interfere with VEGFA signalling. VEGFA resistance in monocytes from T2DM patients can be rescued by either experimental down‐regulation of TGF‐β receptor expression in vitro or by functional blocking of TGF‐β signalling using either a TGF‐β receptor kinase inhibitor or a TGF‐β neutralizing antibody. Our data demonstrate that both T2DM and high‐glucose potentiate the TGF‐β pathway. TGF‐β signalling impairs VEGFR‐mediated responses in T2DM monocytes and in this way contributes to mononuclear cell dysfunction, provide novel insights into T2DM vascular dysfunction.

## 1 INTRODUCTION

Type 2 diabetes mellitus (T2DM) is one of the main risk factors for the development of atherosclerosis. Moreover, the formation of collateral arteries is decreased in patients with T2DM.^1^, ^2^ Monocytes play an important role in vascular biology during angiogenesis and arteriogenesis including the development of collateral vessels following coronary artery occlusion.^3^, ^4^ Any imbalance of number or function of monocytes has detrimental consequences for vascular healing and may lead to cardiovascular pathology such as atherosclerosis.^5^ It has been shown that monocytes from T2DM patients display vascular endothelial growth factor (VEGF)A impaired function,^6^ since they show a reduced migratory response towards VEGFA and placental growth factor (PlGF)‐1, earlier described as VEGFA resistance.^6^, ^7^ Considering the important role of monocytes in arteriogenesis it has been suggested that the decreased formation of collateral vessels in patients with T2DM may be due to an impaired migratory response of monocytes to VEGFA.^7^ Reduced VEGFA responses were shown to be due to increased baseline activity of signalling cascades such as phosphoinositide 3‐kinase (PI3K), extracellular‐signal regulated Kinases (ERK) p44/42 mitogen‐activated protein kinases (MAPK) and p38 MAPK.^6^ Although some of the underlying molecular mechanisms of VEGFA resistance have been characterised,^6^ the exact basis of this remains largely unclear.

Transforming growth factor (TGF)‐β1 is the prototype member of the TGF‐β family of cytokines, which play a crucial role in embryonic development and adult tissue homeostasis.^8^, ^9^ TGF‐β signals via heterotetrameric receptor complexes consisting of type I and type II receptors. Upon ligand binding, the type II receptor phosphorylates and activates the type I receptor which propagates the signal into the cells by activating the SMAD proteins which will regulate gene transcription. In addition to the SMAD pathway, TGF‐β binding to its receptors leads to the activation of the non‐SMAD pathways in a cell‐dependent manner.^8^ TGF‐β signalling plays an important role in angiogenesis and arteriogenesis and perturbation of the TGF‐β pathway leads to (cardio)vascular abnormalities.^9^

Several studies have provided evidence suggesting that the cross‐talk of VEGFA and TGF‐β signalling pathways play an important role in angiogenesis.^8^, ^10^ However, there are no studies on the interplay between the TGF‐β and VEGFA signalling pathways in monocyte function. Interestingly, it has been shown, that the level of TGF‐β1 is elevated in the serum of patients with T2DM^11^ and in the tissue of diabetic mice models.^12^, ^13^, ^14^, ^15^ We have shown previously that despite the fact that monocytes from T2DM patients do not respond to VEGFA and PlGF‐1‐induced chemotaxis, their chemotaxis towards TGF‐β1 is fully functional.^16^ We hypothesized that the TGF‐β pathway interferes with the VEGF pathway in human monocytes contributing to VEGFA resistance.

Here, we show that TGF‐β1 and VEGFA/PlGF‐1 signalling do antagonize each other in inducing monocyte migration. High‐glucose levels enhance TGF‐β responses and by this contribute to mononuclear cell dysfunction and reduced VEGFA‐responsiveness.

## 2 MATERIALS AND METHODS

### 2.1 Cells and reagents

The experiments were performed with peripheral blood human CD14^++^CD16^‐^ monocytes isolated from healthy volunteers and patients. The cells were cultured in RPMI 1640 GlutaMAX (Gibco) or RPMI without glucose (Gibco) supplemented with 5 mmol/L glucose (Sigma‐Aldrich) and 15 mmol/L mannitol (Sigma‐Aldrich) (normal glucose, NG) or 20 mmol/L glucose (high glucose, HG). The medium was supplemented with 10% foetal bovine serum (FBS) (Sigma‐Aldrich) and 1% Penicillin Streptomycin (Gibco). Human VEGFA and PlGF‐1 were obtained from Reliatech and TGF‐β1 from Peprotech. TGF‐β1 kinase receptor inhibitor LY‐364947 was obtained from Tocris Bioscience, DMSO from CalBiochem. The IgG2B Isotype antibody was obtained from R&D system, whereas the TGF‐β neutralizing antibody (a kind gift of Dr E. de Heer, Leiden University Medical Center) was isolated from the 2G7 hybridoma as described before.^17^

### 2.2 Characterization of healthy volunteers and patients

The present study conforms to the principles of the Declaration of Helsinki, and it was approved by the scientific and ethics committee of the University of Münster. Written informed consent was obtained from all subjects. Subjects (a) without T2DM and (b) with T2DM and the patients were recruited in the Department of Cardiology I at the University Hospital Münster. Clinical parameters of the study population are presented in Table 1. Monocytes were also isolated from human blood leukocyte reduction chambers from healthy subjects recruited by the blood bank of the University Hospital Münster.

**TABLE 1:** Patient characteristics

	Without T2DM	With T2DM
	(n = 41)	(n = 24)
Age (mean): years (SD)	58.61 (9.04)	61.5 (6.14)
Sex: n (Male/Female)	28/13	18/6
Hypertension: n (No/Yes)	14/27	3/21
Smoker: n (No/Yes)	28/13	21/3
Dyslipidaemia: n (No/Yes)	24/17	12/12
Obesity: n (No/Yes)	32/9	12/12
Glucose (mean): mg/dL (SD)	104.56 (13.86)	147.67 (36.33)
HbA1c (mean): % (SD)	5.92 (0.41)	7.25 (0.96)

Abbreviations: Hb, haemoglobin; n, number; SD, standard deviation; T2DM, type 2 diabetes mellitus.

### 2.3 Isolation and chemotaxis assays of CD14^++^CD16^‐^ monocytes

Monocytes were isolated as described before.^18^ In brief, blood mononuclear cells were purified via density centrifugation. CD14^++^CD16^‐^ monocytes were isolated from the mononuclear cell fraction with the MACS^®^ monocyte isolation kit II (Miltenyi). The migratory response of the cells towards different ligands was analysed by using a modified Boyden chamber (Neuroprobe) chemotaxis assay as described previously.^16^, ^18^ In brief, monocytes were placed to the upper wells of a 48‐well (Boyden) chemotactic chamber (NeuroProbe) and allowed to migrate towards different concentrations of ligands, which were added to the lower wells of the chamber. Upper and lower wells were separated by a polyethylene terephthalate (PET) membrane (pore size 5 μm Whatman). The cells were allowed to migrate for 90 minutes in a humidified incubator (5.0% CO_2_) at 37°C. Adherent cells on polycarbonate membrane were fixed for 10 minutes using absolute ethanol and stained with Giemsa dye. The non‐migrated cells from the upper side of the membrane were scraped off gently with a cotton bud. Migrated cells were quantified by counting cells in five high‐power fields (20 × primary magnification) of four different wells per condition. Each experimental setting was normalized to each one control before all independent experiments were statistically analysed together.

### 2.4 FACS analysis

Surface expression of the VEGFR1 on CD14^++^CD16^‐^ monocytes was determined by flow cytometric analysis using a FACSCalibur (BD). 0.5 × 10^6^ cells were stained with 0.5 µg Anti‐human VEFR1/Flt‐1‐Phycoerythrin or the corresponding IgG1 isotype control (both R&D system) and used directly for FACS analysis. Events are defined in the forward scatter versus side scatter plot, and afterwards, a gate was defined around the cells. The mean of all stained cells was identified in a single coloured histogram (FACSDiva Version 6.1.3 Software). The relative fluorescence intensity (RFI) was calculated by dividing the mean of VEGFR1 stained cells with the mean of isotype‐stained cells from all samples and used for further analysis.

### 2.5 Protein analysis

Monocytes were cultured in different media with normal or high‐glucose conditions as indicated. Thereafter, cells were processed to lysates and subjected to western blot analysis as described previously.^18^ The intensity of the detected bands was quantified using ImageJ. The ratio of the intensity of the analysed protein to the intensity of detected actin for the corresponding sample was calculated and the values were normalized to control set as 1.

### 2.6 Zymography

Matrix metalloproteinase (MMP)‐2 and MMP‐9 activity was determined by gelatin zymography. Condition medium was mixed with Tris‐Glycine SDS Native sample buffer and loaded/run on a zymogram Tris‐Glycine gelatin gel.^19^ After the separation the gel was washed for 30 minutes with 2,5% Triton and then incubated overnight in Brij solution (16 hours) at 37°C. Gels were stained with Coomassie blue for 60 minutes and after distaining gels were imaged. The band intensity was calculated with ImageJ.

### 2.7 ELISA assay

One millilitre of plasma was collected by centrifugation of 3 mL of patient whole blood sample containing 20 U/mL heparine (Ratiopharm). The total amount of TGF‐β1 in the plasma was measured with the Quantikine^®^ ELISA Immunoassay Kit by the quantitative sandwich enzyme immunoassay technique according to the protocol and measured with the Packard SpectraCount™ (Packard BioScience).

### 2.8 Quantitative real‐time PCR (qRT‐PCR)

RNA isolation was performed with the NucleoSpin^®^ RNAII Kit (Macherey‐Nagel) according to the manufacturer's protocol. RNA was treated with RQ1 DNase (Promega) and used for cDNA synthesis (RevertAid H Minus First Strand cDNA synthesis kit, Thermo Scientific). The qRT‐PCR analysis was done with 2x SYBR Green (Thermo Scientific) in the qPCR‐system Mx3005P (Agilent) or CFX384 Touch (Biorad). As an internal standard, the gene expression of the 18srRNA or GAPDH was used. The analysis was done with the 2(‐Delta Delta C(T)) method.^10^

### 2.9 Statistical analysis

The Statistical analysis and the analysis of the distribution of the data were done in collaboration with the Institute of Biostatistics and Clinical Research (University of Münster). All data are represented as mean with corresponding standard error of the mean (SEM), except the patient characteristics in Table 1 which are represented as mean with corresponding standard deviation (SD). Statistical analysis was performed using SPSS. Parametric distributed data were statistically analysed with One‐way ANOVA with Bonferroni. For non‐parametric distributed data the Wilcoxon matched pair test or the Kruskal‐Wallis test with Dunn's post hoc test for multiple comparisons was used. The generalized linear mixed model (GLMM) was used for the analysis of the results from the chemotaxis assays with monocytes from patients. Differences with values *P* < .05 were considered significant. *P < .05 **P < .01 ***P < .001.

## 3 RESULTS

### 3.1 T2DM enhances monocyte migratory responses towards low concentrations of a TGF‐β1 gradient

**FIGURE 1:**
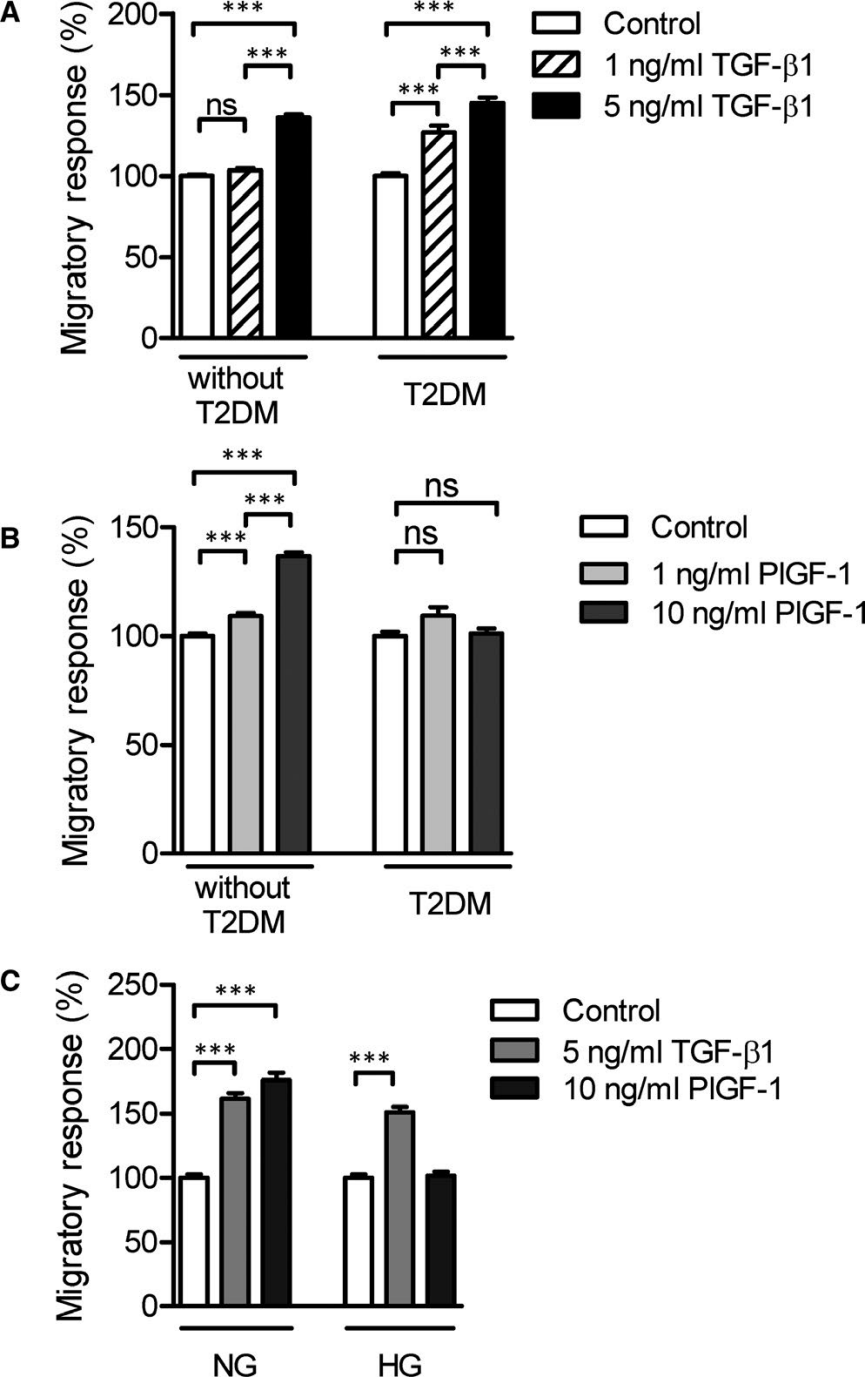
CD14^++^CD16^‐^ monocytes from patients with T2DM show an enhanced migratory response towards TGF‐β1, while T2DM and high glucose show an impaired migratory response towards PlGF‐1. Monocytes were isolated from control subjects without T2DM (n = 20) or patients with T2DM (n = 10). The monocyte chemotactic responses towards TGF‐β1 (A) and PlGF‐1 (B) were analysed. Human CD14^++^CD16^‐^ monocytes (n = 9) were cultured for 24 h under NG or HG conditions, and they were analysed for their chemotactic response towards TGF‐β1 and PlGF‐1 by using the modified Boyden chamber chemotaxis assay for 90 min (C). Data are represented as mean ± SEM. Statistics: GLMM with Gamma regression (A and B) and One‐way ANOVA with Bonferroni (C), *P*‐value: ***<.001. Abbreviations: GLMM, generalized linear mixed model; HG, high glucose; n, number; NG, normal glucose; PlGF, placental growth factor; SEM, standard error of the mean; T2DM, type 2 diabetes mellitus; TGF, transforming growth factor

To characterize the molecular mechanisms of monocyte dysfunction and VEGFA resistance in T2DM we analysed the migratory response of CD14^++^CD16^‐^ monocytes isolated from subjects without T2DM and patients with T2DM towards PlGF‐1 and TGF‐β1. The patient characteristics are shown in Table 1. Although the migratory response of monocytes from patients with T2DM towards PlGF‐1 was impaired (Figure 1B), their migration towards TGF‐β1 was not affected (Figure 1A). Interestingly, monocyte migration towards low concentrations of TGF‐β1 (1 ng/mL) was significantly increased only in T2DM patients compared to non‐T2DM patients (Figure 1A).

### 3.2 High‐glucose concentrations potentiate TGF‐β1‐induced monocyte migration by enhancing TGF‐β signalling

**FIGURE 2:**
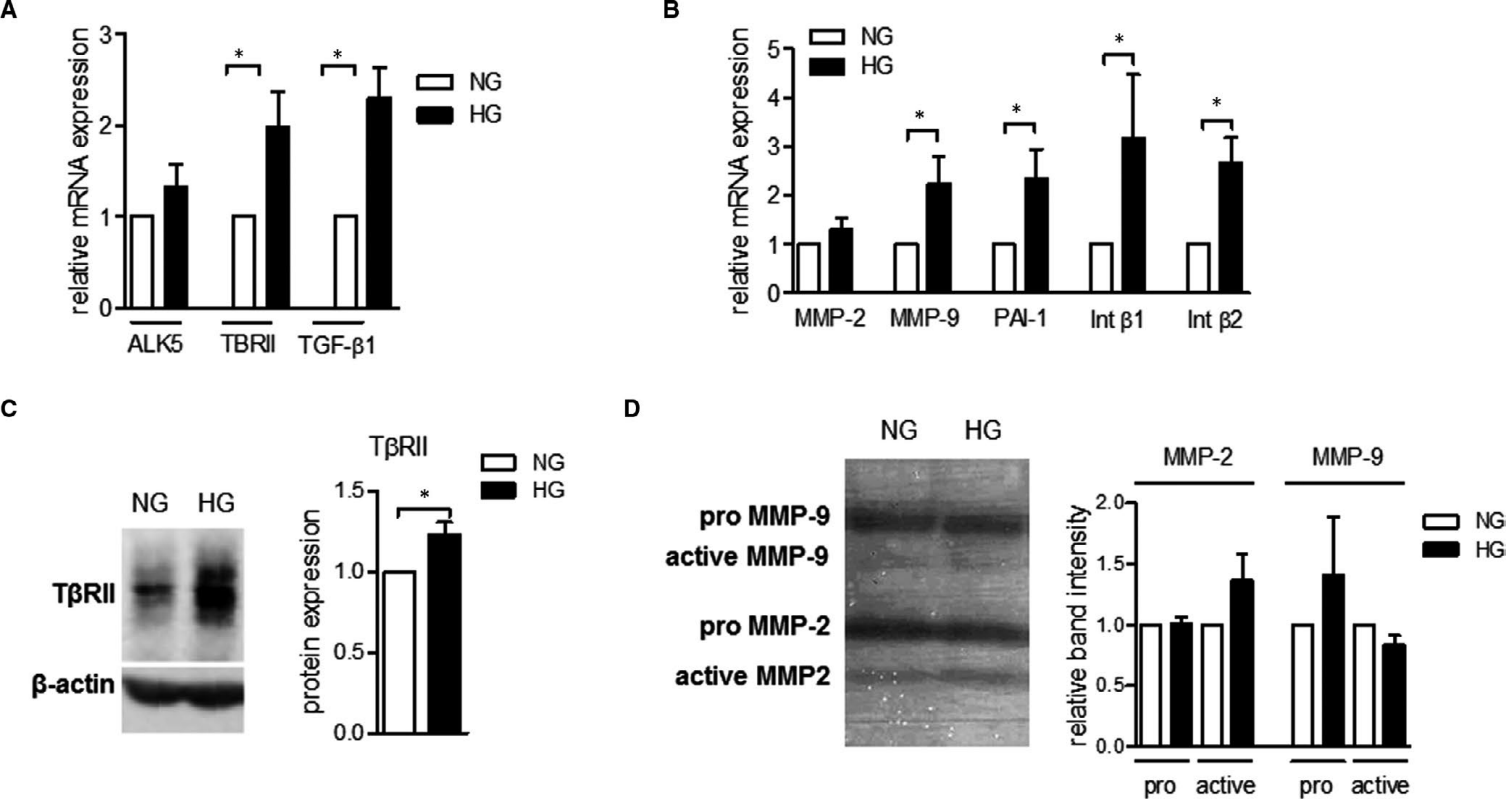
High glucose induces TGF‐β signalling in human CD14^++^CD16^‐^ monocytes. Human monocytes were cultured for 24 h in NG or HG conditions. The mRNA expression of ALK5, TβRII, TGF‐β1 (n = 5) (A) and MMP2, MMP9, PAI‐1, Integrin β1, Integrin β2 and β3 (n = 7) (B) was analysed via qRT‐PCR. The protein expression of TβRII was analysed by Western blot analysis (C). Levels of pro and active MMP‐2 and MMP‐9 were analysed by gelatine zymography (D). Data are represented as mean ± SEM. Statistics: Wilcoxon matched‐pairs signed rank test. *P*‐value: *<.05, ***<.001. Abbreviations: ALK, activin like kinase; HG, high glucose; Int, Integrin; MMP, matrix metalloproteinase; n, number; NG, normal glucose; PAI, plasminogen‐activator inhibitor; SEM, standard error of the mean; TGF, transforming growth factor

Due to the low number of isolated circulating monocytes from patients we employed an in vitro system of high glucose to ‘mimic’ diabetic conditions (high glucose), in order to investigate the molecular mechanisms underlying the enhanced TGF‐β‐induced monocyte responses seen in T2DM. CD14^++^CD16^‐^ monocytes were cultured under normal or high‐glucose conditions and were analysed for their migratory responses. High glucose mitigated PlGF‐1‐induced but not the TGF‐β1‐induced monocyte chemotactic responses in monocytes (Figure 1C). qRT‐PCR expression analysis showed that high glucose significantly induced the gene expression of TGF‐β1 and TβRII in monocytes (Figure 2A). High glucose also induced protein expression of TβRII in primary monocytes (Figure 2C). The expression of the TGF‐β target genes plasminogen‐activator inhibitor (PAI)‐1, MMP‐2, MMP‐9 and Integrin β1 and 2 was enhanced in monocytes cultured under high‐glucose conditions (Figure 2B). Furthermore, zymography analysis revealed that high‐glucose–induced levels of pro‐MMP‐9, however, there was no significant effect on levels of pro‐MMP‐2 in condition media of human monocytes (Figure 2D). These results are in line with the results from gene expression analysis, which demonstrated that high glucose induces mRNA expression of MMP‐9 but not MMP‐2. Of note, high‐glucose conditions induced levels of active MMP‐2 but not levels of active MMP‐9 in condition media of human monocytes cultured under high‐glucose conditions.

**FIGURE 3:**
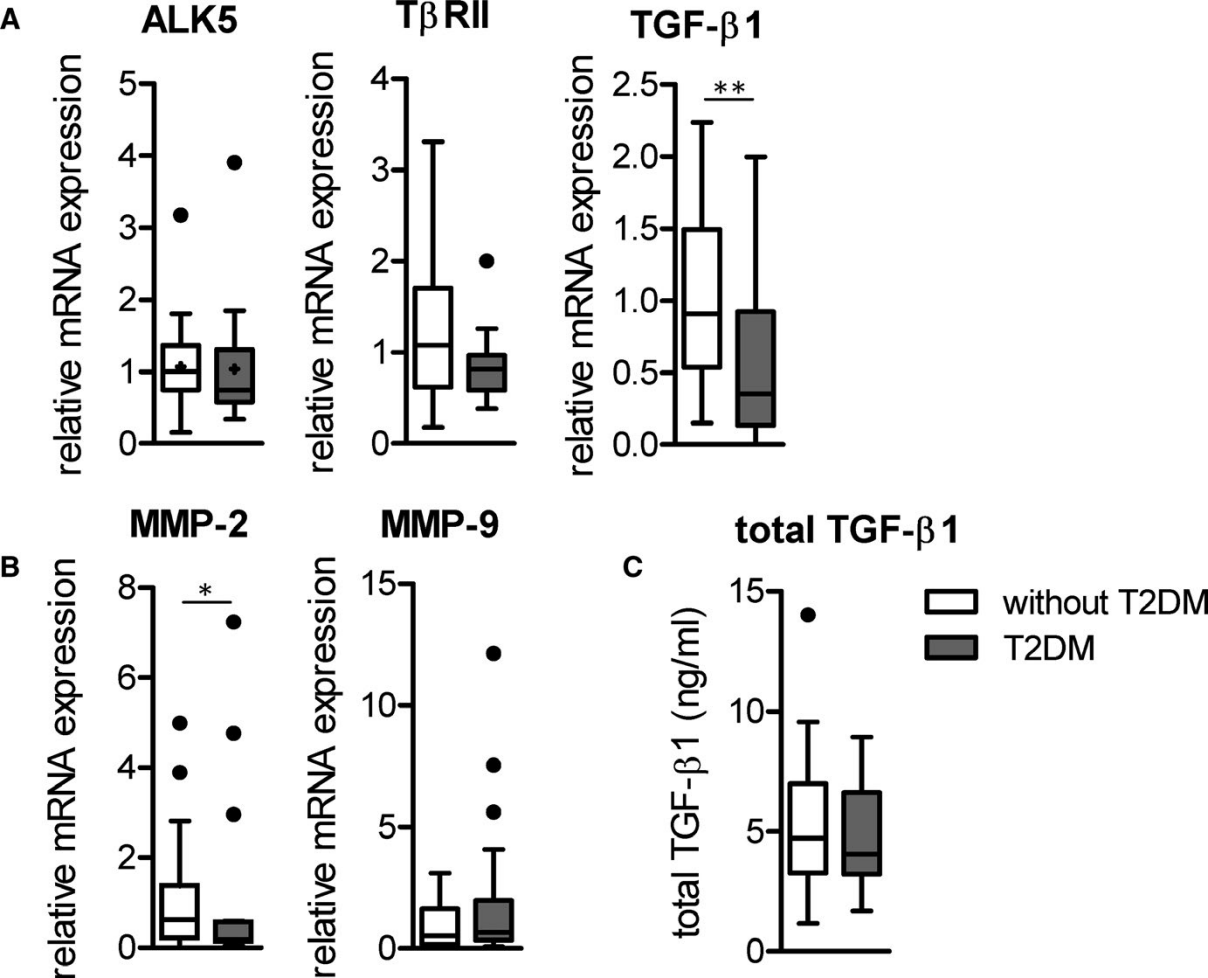
Gene expression of the TβRII, TGF‐β1 and TGF‐β target genes in patient CD14^++^CD16^‐^ monocytes. Monocytes were isolated from the blood of subjects without T2DM (n = 39) or patients with T2DM (n = 18) and the mRNA expression of ALK5, TβRII, TGF‐β1 (A) and MMP2 and MMP9 (B) was analysed via qRT‐PCR. Plasma from subjects without T2DM (n = 41) and with T2DM patients (n = 19) was analysed for the amount of total TGF‐β1 (C). Data are represented as box and whisker and outliers are shown as black points. Statistics: Kruskal‐Wallis test with Dunn; *P*‐value: **<0.01. Abbreviations: ALK, activin like kinase; MMP, matrix metalloproteinase; n, number; SEM, standard error of the mean; T2DM, type 2 diabetes mellitus; TGF, transforming growth factor

Gene expression analysis of TGF‐β signalling molecules and target genes did not show any significant changes in monocytes from T2DM patients compared to non‐T2DM controls (Figure 3A,B). Due to the low number of isolated circulating monocytes from patients analysis of protein levels of the specific molecules was not feasible. Analysis of the total levels of TGF‐β1 in the plasma of both patient groups using a TGF‐β ELISA suggested that there are no differences in the concentration of total TGF‐β1 (Figure 3C).

### 3.3 TGF‐β interferes with VEGFA‐induced monocyte function

**FIGURE 4:**
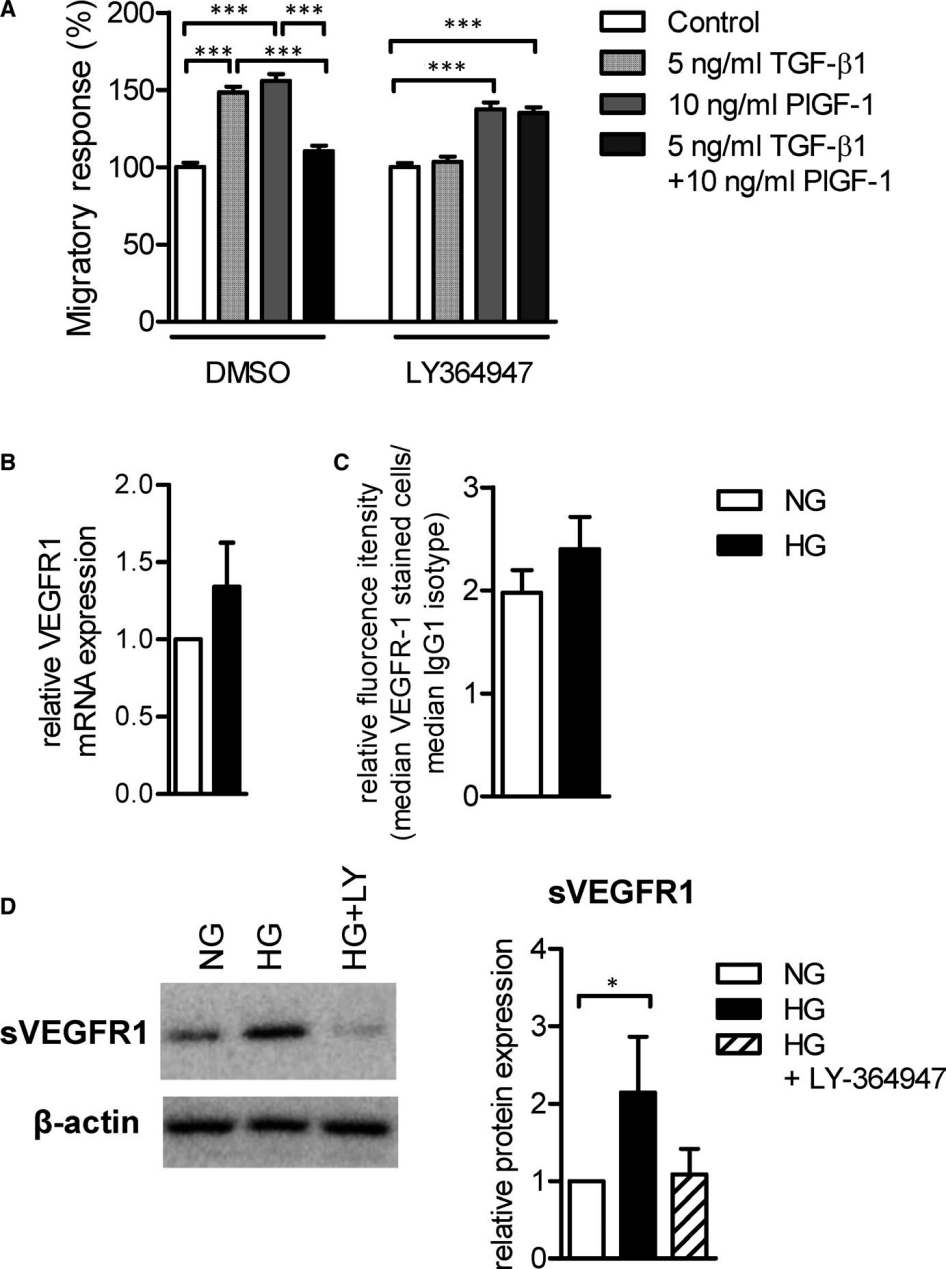
High glucose interferes with PlGF‐1‐induced CD14^++^CD16^‐^ monocyte migration and induces sVEGFR1 expression in a TGF‐β1 manner. Monocytes were incubated for 60 min with DMSO or the ALK5 kinase inhibitor LY364947, and their chemotactic response towards TGF‐β1 and/or PlGF‐1, was analysed by using the modified Boyden chamber chemotaxis assay for 90 min (n = 4) (A). Monocytes were isolated from the blood of healthy individuals and cultured for 24 under NG or HG conditions. The mRNA expression of VEGFR1 was analysed via qRT‐PCR (n = 6) (B). The housekeeping gene *18s rRNA* was used as an internal standard. Furthermore, the protein expression level was determined by flow cytometry and the relative fluorescence intensity (RFI) was calculated by dividing the mean of VEGFR1 stained cells with the mean of isotype stained cells (BD) (n = 3) (C). Monocytes were isolated from the blood of healthy individuals and cultured for 24 h under NG or HG conditions in the presence or absence of the ALK5 kinase inhibitor LY364947 and the protein expression of sVEGFR1 was analysed by Western blot analysis (n = 8) (D). Data are represented as mean ± SEM. Statistic: One‐way ANOVA with Bonferroni (A) and Wilcoxon matched‐pairs signed rank test (B‐D). *P*‐value: *<.05, ***<.001. Abbreviations: (s)VEGFR, (soluble)vascular endothelial growth factor receptor; ALK, activin like kinase; DMSO, dimethyl sulfoxide; HG, high glucose; n, number; NG, normal glucose; PlGF, placental growth factor; SEM, standard error of the mean; TGF, transforming growth factor

Although high glucose and T2DM potentiate TGF‐β‐induced monocyte chemotaxis they interfere with PlGF‐1‐induced monocyte migration. Studies in endothelial cells suggested that TGF‐β1 interferes with VEGFA function.^10^ Thus we further analysed the monocyte migratory responses towards PlGF‐1 and/or TGF‐β1. PlGF‐1 and TGF‐β1‐induced monocyte migration, whereas their combination resulted in no enhanced migratory responses and inhibition of the TGF‐β pathway by using the type I TGF‐β1 receptor kinase inhibitor (ALK5KI) LY364947, reversed this effect (Figure 4A). These results suggest that the two pathways antagonize each other's function in monocytes. Our results suggested that high glucose potentiates TGF‐β‐induced migration by enhancing TβRII expression. Thus we analysed the effects of high glucose on VEGFR1 expression. Although high‐glucose concentrations did not affect gene and protein expression of VEGFR1 (Figure 4B,C) it resulted in increased protein expression of soluble (s)VEGFR1 (Figure 4D), a splice variant of VEGFR1 that acts as a VEGFA/PlGF‐1 trap and inhibits VEGF signalling. Inhibition of endogenous TGF‐β signalling with the ALK5KI LY‐364947, inhibited the high‐glucose induced sVEGFR1 expression (Figure 4D).

### 3.4 Interference with the TGF‐β signalling rescues the impaired PlGF‐1‐induced migratory response of monocytic cells in high‐glucose conditions

To determine whether enhanced TGF‐β signalling has a functional role in the impaired PlGF‐1‐induced monocyte migration in high‐glucose conditions, we studied the effects of inhibiting the TGF‐β pathway.

**FIGURE 5:**
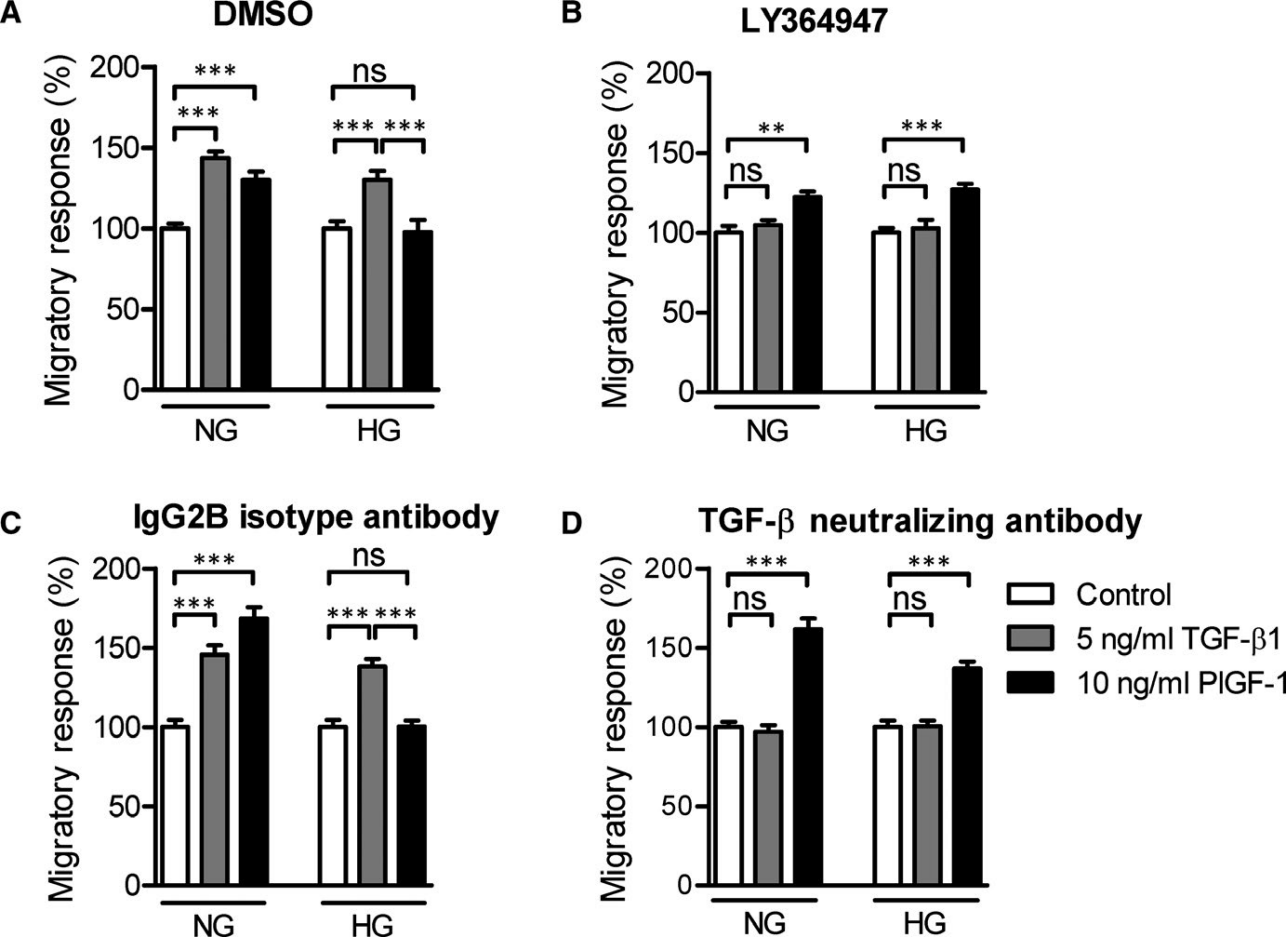
Inhibition of the TGF‐β pathway rescues the impaired migratory response of CD14^++^CD16^‐^ monocytes. Monocytes were isolated from healthy individuals and cultured for 16 h under NG or HG conditions in the presence of DMSO (A) and the ALK5 kinase inhibitor LY‐364947 (B) (n = 4) or 5 µg/mL IgG2B isotype (C) and 5 µg/mL TGF‐β neutralizing antibody (D) (n = 4) and the chemotactic responses towards TGF‐β1 and PlGF‐1 were analysed by using the modified Boyden chamber chemotaxis assay for 90 min. Data are presented as mean ± SEM. One‐way ANOVA with Bonferroni. *P*‐value: **<.01, ***<.001. Abbreviations: ALK, activin like kinase; DMSO, dimethyl sulfoxide; HG, high glucose; n, number; NG, normal glucose; PlGF, placental growth factor; SEM, standard error of the mean; TGF, transforming growth factor

Primary monocytes were cultured under norm or high‐glucose conditions in the presence or absence of the LY364947 type I TGF‐β1 receptor kinase inhibitor. Addition of LY364947 inhibited the TGF‐β1‐induced migration in normal and high‐glucose conditions. LY364947 did not affect PlGF‐1‐induced monocyte migration under normal glucose conditions, however, it rescued the impaired PlGF‐1‐induced monocyte migration in high‐glucose conditions (Figure 5A,B). Inhibition of TGF‐β signalling, by using a neutralizing antibody against TGF‐βs, gave similar results. Addition of the TGF‐β‐neutralizing antibody inhibited TGF‐β1‐induced migration in both normal and high‐glucose conditions. TGF‐β‐neutralizing antibody, although it did not affect PlGF‐1‐induced monocyte migration in normal glucose conditions, fully restored the impaired PlGF‐1‐induced monocyte migration in high‐glucose conditions (Figure 5C,D). Our results suggest that endogenous TGF‐β1 and autocrine TGF‐β signalling has an inhibitory effect on PlGF‐1‐induced monocyte migration in high‐glucose conditions.

### 3.5 Inhibition of TGF‐β signalling rescues the VEGFA resistance in monocytes from T2DM patients

**FIGURE 6:**
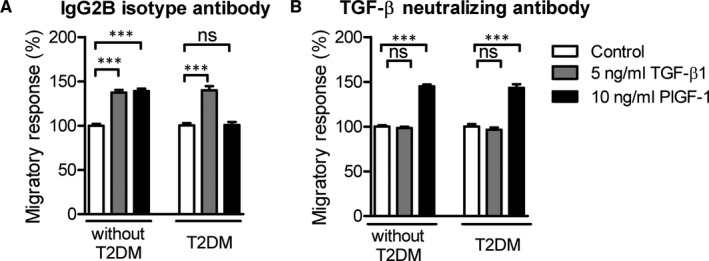
Inhibition of the TGF‐β pathway rescues the impaired migratory response of CD14^++^CD16^‐^ monocytes towards PlGF‐1 in monocytes from T2DM patients. Monocytes from control subjects without T2DM (n = 16) and patients with T2DM (n = 5) were incubated for 16 h with 5 µg/mL of the IgG2B isotype (A) or 5 µg/mL TGF‐β neutralizing antibody (B). The chemotactic response towards TGF‐β1 and PlGF1 was analysed by using the modified Boyden chamber chemotaxis assay for 90 min. Data are presented as mean ± SEM. Statistics: GLMM with Gamma regression. *P*‐value: ***<.001. Abbreviations: GLMM, generalized linear mixed model; n, number; PlGF, placental growth factor; SEM, standard error of the mean; T2DM, type 2 diabetes mellitus; TGF, transforming growth factor

To characterize the role of TGF‐β in mononuclear cell dysfunction in T2DM, we corroborated whether inhibition of endogenous TGF‐β signalling can restore the impaired PlGF‐1 migratory responses of T2DM monocytes. For this monocytes isolated from different patient groups were cultured in the presence of the isotype‐matched control IgG2B or the TGF‐β‐neutralizing antibody and the migratory response towards TGF‐β1 and PlGF‐1 was analysed. Addition of the TGF‐β‐neutralizing antibody blocked the TGF‐β1‐induced chemotaxis of monocytes from both patient groups. Both the control and the TGF‐β neutralizing antibody did not affect PlGF‐1‐induced migratory response of monocytes from patients without T2DM (Figure 6A). Importantly, the TGF‐β neutralizing antibody rescued the impaired PlGF‐1‐induced migration of monocytes from patients with T2DM (Figure 6B). These results illustrate that inhibition of endogenous TGF‐β and autocrine TGF‐β signalling in monocytes from T2DM patients can fully restore their migratory response towards PlGF‐1.

## 4 DISCUSSION

Monocytes from T2DM patients have an impaired migratory response towards VEGFA and PlGF‐1 (ie VEGFA resistance).^6^, ^16^ Here we demonstrate that TGF‐β1‐induced chemotaxis is enhanced in monocytes from patients with T2DM. We show that TGF‐β interferes with VEGFA‐induced monocyte function. We provide mechanistic evidence that high glucose results in enhanced TGF‐β signalling in primary monocytes by inducing gene expression of the TGF‐β1 ligand and the TβRII receptor. Furthermore, we demonstrate that high glucose induces the expression of sVEGFR1 in a TGF‐β‐dependent manner and in this way, it may interfere with PlGF‐1‐induced monocyte chemotaxis. Our results demonstrate that inhibition of endogenous TGF‐β and autocrine TGF‐β signalling restores the mitigated PlGF‐1‐induced migratory responses of monocytes in high‐glucose conditions. Finally, we demonstrate that treatment of monocytes isolated from patients with T2DM with a TGF‐β neutralizing antibody rescued their impaired PlGF‐1‐induced migratory responses. To our knowledge, this is the first study to demonstrate that the TGF‐β pathway contributes to VEGFA resistance and monocyte dysfunction in T2DM.

Our data strongly indicate that high glucose and T2DM lead to enhanced TGF‐β signalling in human monocytes possibly by inducing the expression of the TβRII and TGF‐β1, as suggested by our in vitro data. In line with our results, it was shown that the TGF‐β1 levels are elevated in the serum of T2DM patients,^11^ and in tissue of diabetic mice.^12^, ^13^ It was also shown that high glucose induces TGF‐β in podocytes,^13^ and in mesangial cells.^20^ In contrast to the results from our in vitro system, monocytes from T2DM patients do not shown significant differences in the expression of TGF‐β1, ALK5, TβRII compared to patients without T2DM. These discrepancies are probably due to the low number of patients analysed. Analysis of the TGF‐β1 circulating levels in serum of patients from the MONICA/KORA case cohort, showed that the concentration of the TGF‐β1 ligand in the serum between patients with T2DM and without T2DM did not differ when all patients were summarized together. Interestingly, only after Herder et al^11^ adjusted for the sex, age and survey they found an elevated serum TGF‐β1 concentration in T2DM patients. We cannot exclude the possibility that these discrepancies are due to additional factors that are present in the T2DM patients such as insulin levels, dyslipidemia and metabolic changes, medication, but they cannot be recapitulated in the in vitro system under high‐glucose conditions.

We demonstrate that TGF‐β antagonizes the VEGFA pathway in monocyte migration. We show that high glucose induces the expression of the type II TGF‐β receptor, TβRII and in this way potentiates TGF‐β signalling which in turn interferes with VEGFA‐induced monocyte chemotaxis. In line with this, studies in endothelial cells have provided evidence that TGF‐β interferes both in vitro and in vivo with VEGFA‐induced endothelial cell proliferation, migration and angiogenesis.^9^, ^10^, ^21^ sVEGFR1 is a VEGFA decoy receptor that binds to VEGFA and inhibits its signalling. Moreover, sVEGFR1 was shown to sensitize ECs to pro‐inflammatory cytokines.^22^ We show that high‐glucose conditions induce the expression of sVEGFR1 in a TGF‐β‐dependent manner. Although we could not analyse sVEFR1 in the plasma of the patients included in this study, several studies have shown that sVEGFR1 is increased in plasma of patients with T2DM,^23^, ^24^, ^25^, ^26^ further supporting our in vitro observations.

Further, we show that inhibition of endogenous TGF‐β pathway rescued the impaired VEGFA/PlGF‐1‐induced monocyte chemotaxis in high‐glucose conditions and most importantly in monocytes isolated from patients with T2DM. These results advocate that enhanced endogenous TGF‐β1 and autocrine TGF‐β signalling in T2DM contributes to the VEGFA resistance and monocyte dysfunction in patients with T2DM. Taken together all these findings further suggest that inhibition of TGF‐β might be an attractive target for developing novel therapeutic interventions for the treatment of vascular complications in T2DM. In line with this notion, it was shown recently that metformin, a first‐line antidiabetic drug,^27^ antagonizes TGF‐β1 signalling by blocking the binding of TGF‐β1 to TβRII.^28^ Interestingly, metformin was shown to reduce sVEGFR1 secretion from primary human tissues.^29^ In another study it was shown that inhibition of the TGF‐β/Smad3 pathway in mice protected against diabetes mellitus during high‐fat–induced obesity.^30^, ^31^ Additionally, it was shown that transient inhibition of TGF‐β1 in diabetic peripheral blood CD34^+^ cells ex vivo enhanced their vascular reparative functions in vivo in mice.^32^

Our in vitro culture conditions of human monocytes were designed to create an environment that mimics diabetic conditions. Our in vitro results suggest that increased concentrations of glucose may result in increased levels of TGF‐β expression and potentiation of the TGF‐β signalling which can contribute to mononuclear cell dysfunction and VEGF resistance in T2DM. Our research may serve as a basis for further studies to better characterize the role of TGF‐β1 signalling in mononuclear cell dysfunction and cardiovascular complications in patients with T2DM.

In summary our data indicate that high glucose and T2DM result in increased TGF‐β signalling which interferes with VEGFA‐induced monocyte migratory responses and contributes to monocyte dysfunction. Moreover, inhibition of TGF‐β signalling restores mononuclear cell responses in monocytes from T2DM patients. Our results suggest that TGF‐β signalling affects monocyte function and thereby contributes to vascular complications in patients with T2DM.

## CONFICT OF INTEREST

The authors ML and EP have no conflicts of interest. LM reports other from Bayer Vital, outside the submitted work. JW reports personal fees and non‐financial support from Biotronik, personal fees from Akzea, personal fees from Bayer Vital, personal fees and non‐financial support from Boehringer Ingelheim, personal fees and non‐financial support from Daiichi‐Sankyo, personal fees from MSD, personal fees from Berlin‐Chemie, personal fees from Siemens Healthineers, outside the submitted work.

## AUTHOR CONTRIBUTION

**Lena‐Maria Makowski:** Data curation (lead); Formal analysis (lead); Investigation (lead); Methodology (lead); Writing‐original draft (equal); Writing‐review & editing (equal). **Merle Leffers:** Investigation (supporting); Methodology (supporting); Writing‐review & editing (supporting). **Johannes Waltenberger:** Funding acquisition (equal); Writing‐review & editing (lead). **EVANGELIA PARDALI:** Conceptualization (lead); Data curation (supporting); Formal analysis (supporting); Funding acquisition (equal); Project administration (lead); Supervision (lead); Writing‐original draft (lead); Writing‐review & editing (lead).

## Data Availability

The data that support the findings of this study are available from the corresponding authors upon reasonable request.
